# Prenatal factors shaping adolescent cardiovascular function and cardiometabolic risk trajectories

**DOI:** 10.3389/fped.2026.1933223

**Published:** 2026-09-14

**Authors:** Zhuoyuan A. Li, Webb A. Smith, Aaron Walsh, Lauren Sims Taylor, Qi Zhao

**Affiliations:** 1 College of Medicine, University of Tennessee Health Science Center, Memphis, TN, United States; 2 Department of Pediatrics, College of Medicine, University of Tennessee Health Science Center, Memphis, TN, United States; 3 Division of Pediatric Cardiology, Department of Pediatrics, College of Medicine, University of Tennessee Health Science Center, Memphis, TN, United States; 4 Department of Preventive Medicine, College of Medicine, University of Tennessee Health Science Center, Memphis, TN, United States

**Keywords:** adolescence, cardiovascular, development, pregnancy, prenatal factors

## Abstract

Cardiovascular disease remains the leading cause of mortality worldwide, with early risk markers, including elevated blood pressure, arterial stiffness, and adverse lipid profiles, often emerging during adolescence. These phenotypes are clinically important, as adolescent cardiovascular risk strongly predicts adult subclinical atherosclerosis and future cardiovascular events. Increasing evidence from developmental origins of health and disease (DOHaD) research suggests that cardiovascular outcomes in adolescence are associated, in part, with prenatal exposures. This review synthesizes current evidence linking prenatal factors to cardiovascular outcomes in adolescence and examines the biological mechanisms that may mediate these associations. A structured literature search of PubMed from database inception through March 14, 2026 was conducted, including peer-reviewed studies published in English that examined associations between prenatal exposures and cardiovascular outcomes in adolescent populations. Growth-related metrics, particularly low birth weight and preterm birth, emerged as the most consistent correlates of elevated blood pressure and adverse structural phenotypes. Maternal preeclampsia and gestational hypertension were associated with altered offspring cardiac structure and endocrine regulation. Maternal obesity and diabetes were correlated with adverse cardiometabolic outcomes, though these associations were largely mediated by offspring adiposity, suggesting a prominent role for shared genetic and postnatal environmental factors. Prenatal nutritional interventions showed limited effects on cardiovascular outcomes, while environmental exposures such as maternal smoking and chemical pollutants were associated with increased risk, potentially through oxidative stress and disrupted organogenesis. Common mechanistic pathways across exposure categories included impaired nephrogenesis, hypothalamic-pituitary-adrenal axis dysregulation, and disrupted autonomic regulation. Despite these advances, significant knowledge gaps remain in fully understanding the contribution of prenatal factors to cardiovascular health during adolescence. Further studies utilizing state-of-the-art technologies to better characterize prenatal exposures, including metabolomic, exposomic, and other omics-based approaches, are needed to clarify the biological pathways linking the prenatal environment to adolescent cardiovascular risk and to inform earlier risk identification and preventive strategies.

## 1 Introduction

Cardiovascular disease (CVD) remains the leading cause of death worldwide, accounting for an estimated 19.8 million deaths in 2022 (1). A substantial share of this burden is attributable to modifiable cardiometabolic risk, particularly elevated blood pressure and atherogenic lipid profiles, which contribute to atherosclerosis, stroke, and other downstream vascular outcomes (2). Because CVD develops over decades, identifying when adverse cardiovascular physiology first emerges and what shapes it is central to prevention (3).

Although clinical events typically occur in adulthood, measurable cardiovascular outcomes are already present in adolescence, including blood pressure, heart rate, arterial stiffness, and circulating lipids (4). Recent research suggests pediatric hypertension prevalence has increased markedly over time (5, 6), and population data indicate that a sizable proportion of youth have at least one lipid abnormality (7). These adolescent measures matter because they persist; blood pressure shows clear tracking from childhood and adolescence into adulthood (8), and lipid levels similarly track across the life course (9, 10). Consistent with this, adolescent risk factor profiles can predict adult subclinical atherosclerosis, such as carotid intima-media thickness (11), and are linked to later clinical cardiovascular events (12, 13).

While adolescent cardiovascular outcomes are influenced by postnatal lifestyle and inherited susceptibility, these factors do not fully explain individual differences. Growing evidence supports a developmental origins framework, termed Developmental Origins of Health and Disease (DOHaD), in which prenatal exposures shape later cardiovascular risk (14). Epidemiologic research links fetal growth to later blood pressure, with lower birth weight associated with higher systolic blood pressure (SBP) in later life (15). Gestational age also appears important: individuals born preterm show modestly higher blood pressure later and, in adolescent cohorts, may demonstrate sex-specific elevations in blood pressure and more atherogenic lipid patterns (16, 17). Maternal cardiometabolic conditions during pregnancy similarly relate to offspring cardiovascular outcomes; exposure to hypertensive disorders of pregnancy is associated with higher offspring blood pressure and greater hypertension risk (18, 19), and maternal diabetes and gestational diabetes have been linked to higher offspring blood pressure and increased risk of hypertension (20, 21). Maternal adiposity and overnutrition are likewise associated with higher offspring blood pressure across studies and meta-analyses (22, 23). In this review, we synthesize evidence on how prenatal factors relate to adolescent cardiovascular function and discuss mechanisms that may connect the prenatal environment to risk trajectories across the life course (14, 24).

## 2 Methods

This narrative review was conducted using a structured literature search of PubMed from database inception to March 14, 2026. Search terms included keywords related to prenatal exposures (e.g., fetal programming, developmental origins, *in utero* exposure, maternal exposure, DOHaD), cardiovascular outcomes (e.g., blood pressure, arterial stiffness, pulse wave velocity, vascular function, cardiac remodeling), and the adolescent population. Both MeSH terms and free-text searches were used to maximize retrieval. The complete search string is available in Supplementary Table 1.

Titles and abstracts were screened for relevance, followed by a full-text review of selected articles for inclusion. Studies were included if they were peer-reviewed, published in English, and examined associations between prenatal exposures and cardiovascular or cardiometabolic outcomes in adolescent populations (ages 10–19). Eligible prenatal exposures comprised birth weight, preterm birth, maternal cardiometabolic conditions, nutritional interventions, and environmental exposures. Exposures that did not fall into these categories were retained and synthesized under an “Other” heading. Studies were excluded if the outcomes were reported without adolescent specific estimates, the exposure was postnatal, or the full text was not available. The search returned 393 records, of which 83 met eligibility criteria for this review.

## 3 Results and discussion

### 3.1 Cardiovascular health measures during adolescence

Across the included studies, adolescent cardiovascular health outcomes cluster into four main measurement categories: blood pressure, cardiometabolic risk, cardiac function, and arterial stiffness (Table 1). Blood pressure outcomes are the most frequently captured, spanning clinical hypertension, SBP and diastolic blood pressure (DBP), and related hemodynamic indices such as mean arterial pressure, plus more context-specific measures like blood pressure response to stress and post-exercise blood pressure. Cardiometabolic outcomes emphasize adolescent metabolic risk profiling, most commonly body mass index (BMI), overweight/obesity, lipids [triglycerides, cholesterol, low-density lipoprotein cholesterol (LDL-c) and broader lipid profiles], and glycemic and insulin measures (fasting insulin, insulin resistance, blood glucose, fasting glucose, and glucose metabolism), with some inclusion of neuroendocrine stress markers [salivary cortisol, plasma adrenocorticotropic hormone (ACTH), plasma cortisol] and broader syndromic classifications like metabolic syndrome. More specialized measures appear less often but include cardiac measures [left ventricular mass, heart rate, heart rate variability, systolic and diastolic function, ventricular output and stroke volume, corrected QT (QTc) interval] and vascular stiffness and structure markers [pulse wave velocity (PWV), plus aortic measures and carotid intima-media thickness]. Finally, a subset of studies summarizes risk using composite indices such as the Pathobiological Determinants of Atherosclerosis in Youth (PDAY) risk score, cardiometabolic risk scores, overall cardiovascular health risk, and a global cardiovascular health score.

**Table 1:** Summary of cardiovascular health measures studied during adolescence.

Outcome category	Outcome measurements
Blood pressure	hypertension (25–37), SBP (17, 27, 28, 31, 34, 36, 38–70), DBP (17, 27, 28, 36, 39, 41, 44, 46, 48, 50, 51, 56, 57, 59, 62, 63, 65, 69), blood pressure (41, 65, 71–83), mean arterial pressure (64), blood pressure response to stress (84), blood pressure after exercise (85)
Cardiac function	left ventricular mass (35), resting heart rate (76, 86), end-diastolic vessel diameters (86), heart rate (59, 72, 76, 84, 87), cardiosympathetic stress responses (88), wall thickness (89), left ventricular end-diastolic volume (89), heart rate variability (90), systolic function (91), diastolic function (91, 92), cardiac structure (91, 93), cardiac function (92, 93), right ventricle cardiac output (94), stroke volume (94), QTc interval (95)
Arterial stiffness	PWV (30, 42, 45, 72, 79, 80), arterial stiffness (75), aortic narrowing (41), augmentation pressure (96), aortic distensibility (73), aortic PWV (73)
Cardiometabolic risk factors	BMI (34, 46, 48, 50, 52, 58, 64, 71, 74, 97), triglycerides (64, 78), lipid profile (49, 50, 57, 74, 75, 97, 98), cholesterol (17, 27, 40, 46, 97), salivary cortisol levels (84), fasting insulin (50), insulin resistance (52, 99), LDL-c (17), plasma triglyceride (52), blood glucose (52), insulin (52), plasma ACTH (47), plasma cortisol (47), metabolic syndrome (100), dysmetabolic traits (101), overweight/obesity (26), glucose metabolism (97), fasting blood glucose (46), carotid intima-media thickness (30), cardiovascular health score (102)
Other	PDAY risk score (103), cardiometabolic risk scores (104), cardiovascular health risk (105), cardiometabolic risk z-scores (106)

### 3.2 Prenatal factors associated with cardiovascular health during adolescence

Prenatal factors that have been investigated for associations with cardiovascular health during adolescence were categorized into eight main categories: birth weight, preterm birth, hypertensive disorders of pregnancy, maternal obesity, maternal diabetes, nutrition, environmental exposures, and other factors (Table 2). Birth weight related exposures are the most frequently captured, spanning birth weight, intrauterine growth restriction (IUGR), small and large for gestational age (SGA and LGA), and fetal growth velocity. Preterm birth is the second most represented category. Hypertensive disorders of pregnancy (HDP) emphasize the maternal hemodynamic environment, most commonly preeclampsia (PE) and gestational hypertension (GH), with some studies examining hypertensive disorders of pregnancy as a broader classification. Maternal metabolic exposures also feature prominently, with maternal obesity studies capturing pre-pregnancy BMI, obesity, and maternal adiposity measures, and maternal diabetes studies encompassing gestational diabetes, maternal diabetes, cord blood insulin, and glycosuria. Nutritional exposures appear less often but include supplemental nutrition, fish oil supplementation, vitamin D, and calcium supplementation. Environmental exposures constitute a diverse category, spanning maternal smoking, persistent organic pollutants, per- and polyfluoroalkyl substances (PFAS), heavy metals (antimony, arsenic, cadmium, cobalt, lead, magnesium, molybdenum, selenium), mercury, maternal noise exposure, phthalates, and phenols. Finally, a subset of studies examines less commonly studied exposures such as antenatal corticosteroid therapy, prenatal cocaine exposure, perinatal HIV, maternal hyperemesis gravidarum, race and maternal substance abuse, and accelerated epigenetic age at birth. More details regarding each category are provided in the following section.

**Table 2:** Summary of prenatal factors studied in relation to adolescent cardiovascular health.

Exposure category	Prenatal exposure
Birth weight	high birthweight (25, 39, 62, 67, 101), low birthweight (36, 38, 39, 71, 87, 98, 101), IUGR (65, 86, 92), very low birthweight (65), growth restriction (37, 86), birth weight (82, 91), fetal growth velocity (40), SGA (40, 99), LGA (25, 99), extremely low birthweight (95)
Preterm birth	preterm birth (17, 41, 42, 44–46, 69, 72, 85, 90, 92–94), extreme preterm birth (43), very preterm birth (73)
Hypertensive disorders of pregnancy	preeclampsia (27, 28, 34, 47, 48, 54, 66, 68, 89, 102), gestational hypertension (26–28, 35, 47, 89, 102), hypertensive disorders of pregnancy (70, 105)
Maternal obesity	maternal pre-pregnancy BMI (29, 39, 52, 62), maternal arm fat area (98), maternal obesity in extreme preterm birth (53), pre-pregnancy obesity (102)
Maternal diabetes	maternal diabetes (49, 64, 74, 81, 88, 101), cord blood insulin (51, 96), gestational diabetes (50, 105), glycosuria (50)
Nutrition	nutrition supplementation (75, 77), fish oil supplementation (76), maternal vitamin D (78), vitamin D (54, 78, 79), calcium supplementation (55)
Environmental exposures	maternal smoking (30, 41, 56, 83, 100), mercury (59), maternal noise exposure (33), antimony (32), arsenic (32), cadmium (32), cobalt (32), lead (32), magnesium (32), molybdenum (32), selenium (32), PFAS (31, 104), persistent organic pollutants (57, 58, 97), phthalates (80), phenols (80)
Other	antenatal corticosteroid therapy (63), cocaine (84), perinatal HIV (103), maternal hyperemesis gravidarum (61), race and maternal substance abuse (60), accelerated epigenetic age at birth (106)

### 3.3 Birth weight

Current research indicates that birth weight is a significant predictor of cardiovascular outcomes in adolescence, though the nature of this relationship varies across the weight spectrum. Lower birth weight is associated with increased SBP in adolescents aged 12 to 17 (38). However, this association is frequently masked by body weight; SGA adolescents typically maintain lower body mass than their AGA counterparts, and when adjusting for current body weight, they exhibit blood pressure levels disproportionately high relative to their size (39, 71). SGA is also associated with early markers of metabolic syndrome, including insulin resistance and an atherogenic lipid profile (40). High birth weight (>4,000 g) and being LGA are associated with high blood pressure and an increased risk of developing metabolic syndrome in childhood and adolescence (25).

Abnormal fetal growth also leaves lasting structural imprints on the cardiovascular system. The most persistent of these are observed in the vasculature, where a decreased diameter of large elastic arteries, such as the abdominal aorta, remains evident into young adulthood (86). Fetal growth restriction is further linked to poorer global cardiac function, characterized by a lower shortening fraction, worse E/e' ratio, and a longer myocardial performance index compared to non-growth restricted peers (92). At the other end of the spectrum, higher birth weight adjusted for gestational age has been associated with small increases in left ventricular mass index and differences in E/A and E/e' ratios, though these effects appear modest (91).

The development of cardiovascular dysfunction in adolescents born with low birth weight has been attributed to alterations in vascular and renal structures programmed *in utero* (38, 39). In animal models, placental insufficiency decreases fetal volume blood flow, and the subsequent decreases in shear stress may influence the matrix metalloproteinase system, resulting in hypotrophic vascular remodeling (86). Impaired elastin deposition, demonstrated in rat models, has been proposed to underlie the decrease in central elastic artery diameter in humans that persists after correcting for body surface area (86). These vascular changes are often accompanied by impaired nephrogenesis (38); altered renal structure and function are proposed as primary drivers for programmed hypertension, as they impair long-term pressure regulation (39). Endocrine programming may act in parallel (38, 39); excess fetal exposure to glucocorticoids is a proposed mechanism as it may alter the hypothalamic-pituitary-adrenal (HPA) axis and promote hypertension (39), and fetal growth restriction is associated with a higher resting heart rate in adolescence and young adulthood, indicating a programmed shift towards sympathetic dominance (86).

In contrast, elevated blood pressure in adolescents born with high birth weight appears to be driven less by structural deficits than by an increased risk of obesity. Obesity in turn, contributes to hormonal perturbations, renal structural damage, and altered metabolic and autonomic regulation, including increased sympathetic dominance, activation of the renin-angiotensin-aldosterone system, and altered vasodilator responses (25, 39).

Metabolic traits in adolescence further illustrate how birth weight interacts with postnatal growth to shape cardiovascular risk. Adolescents born SGA or LGA frequently exhibit greater insulin resistance than those born AGA (99). In the case of SGA adolescents, this metabolic phenotype is strongly associated with rapid postnatal catch-up growth, which has been hypothesized to involve tissue-specific increases in glucose uptake, particularly in adipose tissue (40). Importantly, the associations between SGA and these early markers of metabolic syndrome are substantially attenuated when adjusting for body fat percentage and trunk-fat percentage, suggesting that central adiposity, rather than body weight alone, is a key mediator of the metabolic consequences of low birth weight (40). Thus, while the mechanisms differ across the birth weight spectrum, both low and high birth weight converge on elevated adolescent cardiovascular risk, the magnitude of which is ultimately shaped by postnatal growth and adiposity.

### 3.4 Preterm birth

Overall, preterm birth is associated with adolescent cardiovascular differences, though consistency varies by outcome. The most reproducible finding is higher blood pressure. Most cohorts reported higher SBP and/or DBP among adolescents born preterm, including ambulatory elevations in extremely preterm (<28 weeks) individuals and higher systolic responses to exercise at lower gestational ages (17, 41–46, 72, 85). In contrast, associations with arterial structure and stiffness are less consistent. The literature on arterial stiffness is conflicting, though these inconsistencies may be attributable to methodological differences. Of the two studies reporting elevated stiffness, one measured carotid-radial PWV (42), an upper-limb muscular segment whose determinants of stiffness differ from those of the aorta, while the other measured carotid–femoral PWV, the reference-standard aortic index (45). Neither of the two null studies used the same approach as either: one assessed aortic PWV and distensibility directly by magnetic resonance imaging and found no group differences despite enrichment for fetal growth restriction (73), and an earlier study derived aortic stiffness from ultrasound diameter and cuff blood pressure, reporting higher blood pressure alongside lower calculated stiffness (72). Findings on aortic narrowing are partially concordant but sensitive to adjustment. Very preterm birth was associated with a narrower thoracic (16%) and abdominal (19%) aorta on MRI, after adjustment for body surface area (41). A separate study likewise found visibly smaller aortas in adolescents born preterm with fetal growth restriction, but this difference did not persist after adjustment for body surface area (73). Whether aortic narrowing reflects a disproportionate structural deficit or scales with attained body size remains unresolved. Across both outcomes, all the contributing studies enrolled fewer than 60 participants per group and are therefore powered only for large effects. Definitive conclusions about arterial stiffening or aortic narrowing in adolescents born preterm therefore cannot yet be drawn, and the elevated blood pressure observed in these cohorts may be more consistently attributable to increased peripheral resistance than altered large artery structure.

Beyond vascular outcomes, very preterm (<32 weeks) adolescents showed evidence of worse diastolic and global ventricular function, and separate work reported smaller biventricular chamber size without hypertrophy, consistent with a persistent premature heart phenotype (92, 93). Effects on cardiometabolic outcomes were less consistent and appear to be modulated by gestational age and sex. The EVA-Tyrol study found no differences in BMI, total cholesterol, or fasting glucose in adolescents born preterm (mean gestational age 34.6) (46), whereas the Northern Finland Birth Cohort 1986 reported a more atherogenic lipid profile in boys born early preterm (<34 weeks) relative to term-born (>36 weeks), with weaker and mostly non-significant differences among late preterm (<36 weeks) adolescents (17). These findings suggest a dose- and sex-dependent relationship, which the authors of the Northern Finland Birth Cohort Study proposed to be mediated by earlier onset of puberty in preterm children.

Mechanistically, the literature supports several complementary pathways. First, preterm birth may interrupt late-gestation vascular maturation by abruptly transitioning from placental to postnatal circulation during a sensitive growth period. Cessation of placental flow reduces aortic blood flow and exposes the circulation to higher systemic pressures. Vascular growth under these conditions may result in reduced aortic diameter and higher peripheral resistance (41, 72), as well as impaired elastin synthesis and arterial wall remodeling (85). The heart itself may be similarly affected by the premature transition to extrauterine life. Preclinical models suggest the relative hyperoxia after birth is associated with increased reactive oxygen species signaling, which can accelerate terminal cardiomyocyte cell cycle arrest and produce persistent differences in chamber size and function (93).

Renal and neurohormonal mechanisms may also contribute to the elevated blood pressure seen in adolescents born preterm. Nephrogenesis continues until approximately 36 weeks of gestation, and its interruption potentially results in reduced nephron number and maturity (46). One adolescent cohort reported higher SBP and DBP alongside lower estimated glomerular filtration rate, with proposed mechanisms including reduced nephron endowment, altered uric acid metabolism, and changes in the renin-angiotensin system (44). However, these findings are not universal. Another study found normal kidney function in its preterm birth cohort and limited adverse effect of very or extremely preterm birth on kidney function and blood pressure (73). The relevance of renal pathways may therefore depend on the degree of prematurity and the presence of modifying factors such as overweight or obesity (44).

Maternal and social co-exposures may also confound or compound risk. In the MRI cohort described earlier, maternal smoking, for example, was more common among mothers who delivered preterm and was hypothesized to be associated with increased offspring blood pressure (73). However, this study had under 30 participants in each group, and a substantially larger Finnish birth cohort was better positioned to test it: among 6,642 adolescents, of whom 79 were born before 34 weeks and 238 at 34 to 36 weeks, associations between preterm birth and blood pressure persisted after adjustment for maternal smoking (17). Other factors such as parental education have also been considered, though parental education did not differ appreciably by preterm status in either cohort, and adjustment for it did not attenuate the association in the Finnish study. Additionally, downstream consequences of prematurity may influence blood pressure trajectories via chronic stress, as preterm individuals may have higher rates of anxiety and learning difficulties (43).

Taken together, preterm birth is most consistently linked to higher adolescent blood pressure whereas evidence for arterial stiffening varies by cohort. The underlying risk likely reflects the interaction of disrupted vascular and cardiac development, variable renal impairment, and clustered maternal and social exposures (41, 44, 72, 73, 85).

### 3.5 Hypertensive disorders of pregnancy

Exposure to HDP is consistently linked to adverse cardiovascular outcomes in adolescent offspring. Adolescents born to mothers with GH or PE were found to exhibit significantly higher odds of high blood pressure compared to those born to normotensive mothers (26–28, 47). These changes are often accompanied by increased risks of overweight and obesity, with exposed adolescents showing wider waist circumferences and higher mean body mass index (26, 48). Evidence regarding adolescent lipid profiles is weaker. Exposure to maternal GH has been linked to a tendency toward higher fasting cholesterol and apolipoprotein B (28), though this is a directional trend rather than significant association, while a UK cohort reported no significant associations between HDP and fasting lipids, glucose, or insulin levels (27). However, both cohorts were substantially better powered for blood pressure than for metabolic outcomes, and in the UK cohort fasting lipids were available in roughly two-thirds of the offspring with blood pressure data. The available data therefore favor a blood pressure-specific association rather than a generalized cardiometabolic disturbance.

Beyond systemic blood pressure and metabolic markers, maternal hypertension appears to influence the development of the fetal heart, leading to a distinct cardiac structure in adolescence. Offspring exposed to PE often demonstrate a pattern approaching concentric remodeling characterized by a greater relative wall thickness and lower left ventricular end-diastolic volume, while exposure to maternal GH is associated with increased relative wall thickness; however, global cardiac function is not affected (89). Interestingly, mothers with HDP showed wall thickness changes similar to those found in their adolescent offspring. The authors hypothesized that inherited differences in heart structure that predispose to HDP may be a confounding factor in this relationship.

The mechanisms linking HDP to offspring cardiovascular outcomes differ slightly between PE and GH. Lower maternal cortisol levels in late gestation associated with PE may induce compensatory upregulation of the fetus' HPA axis, which has been proposed to underlie the elevated plasma cortisol and ACTH in adolescence (47). Pro-inflammatory cytokines such as IL-6 and TNF-α are elevated in PE and can cross the placenta to reach fetal circulation, where they may further stimulate the HPA axis. The resulting excess of glucocorticoids may contribute to blood pressure dysregulation; in rat models, glucocorticoids increase sodium and calcium uptake within vascular muscle cells and enhance the vasoconstrictive actions of angiotensin II and noradrenaline (28).

GH is not typically associated with elevated basal HPA activity, but it shares several pathways with PE that influence long-term cardiovascular regulation (47). In both conditions, *in utero* exposure to maternal hypertension appears to affect cardiac development, leading to altered structure in adolescence (89). Both conditions also expose the fetus to dysregulated maternal and fetal glucocorticoids, which can impair the maturation of organs responsible for blood pressure control, including the kidneys, as well as the sympathoadrenal system that mediates autonomic cardiovascular regulation later in life (28). Where the two conditions diverge is in their endocrine signatures: while PE is associated with compensatory HPA axis upregulation, GH offspring show a suppressed ACTH-to-cortisol ratio (47).

Crucially, HDP are a driver of restricted intrauterine growth; the resulting SGA status can trigger rapid postnatal catch-up growth, which, as discussed in the section on birth weight, acts as an independent risk factor for the development of metabolic and cardiovascular dysfunction in the offspring (48).

### 3.6 Maternal diabetes

Exposure to maternal gestational diabetes mellitus (GDM), type 1 diabetes mellitus (T1DM), and glycosuria have been examined in relation to blood pressure and cardiometabolic risk factors, with findings differing across studies. In the EPOCH cohort, in-utero exposure to GDM (*n* = 92) was associated with higher SBP (4.50 mmHg, 1.90–7.10) in boys and elevated total cholesterol (0.38 mmol/L, 0.16–0.61) and LDL-c (0.34 mmol/L, 0.14–0.53) in girls (49). The patterns of adjustment in the GDM cohort suggest that blood pressure and lipid outcomes are sex-specific and arise through different pathways; the elevated SBP in boys was attenuated to non-significance after adjustment for BMI but robust to adjustment for GDM treatment, suggesting a mechanism mediated by offspring adiposity rather than maternal hyperglycemia (49). The lipid patterns in girls showed the reverse; they were robust to BMI adjustment and attenuated after adjustment for GDM treatment, consistent with a hyperglycemia mediated mechanism. The authors of this cohort study proposed that the sex-specific differences arose outside the intrauterine environment. The confinement of the adverse lipid profile to girls may reflect residual confounding by pubertal tempo, as GDM-exposed girls have been reported to enter puberty earlier than boys and earlier puberty is accompanied by more rapid increases in total cholesterol and LDL-c. The elevated SBP in boys is hypothesized to instead reflect the steeper rise and greater variability in blood pressure through adolescence in males (49).

In a separate cross-sectional study of 103 offspring of mothers with T1DM and 98 unexposed offspring, maternal T1DM was associated with elevated blood pressure, BMI, total cholesterol, LDL-c, triglyceride, estimated glomerular filtration rate, and microalbuminuria (74). The broader set of associations with T1DM is consistent with a more severe exposure spanning organogenesis and the whole of gestation rather than beginning in the late second trimester. This however, rests on a single study of each exposure, and the T1DM study could not quantify antenatal hyperglycemia beyond self-reported insulin treatment, so a severity gradient across subtypes remains plausible but untested.

A second cohort, the ALSPAC cohort, examined pre-existing diabetes, (*n* = 23), GDM (*n* = 27), and glycosuria (*n* = 154) and found little evidence of association with offspring SBP, DBP, or lipids, though they were associated with increased BMI and fat mass z scores (50). Though the ALSPAC cohort contradicts the other two studies, it had small group sizes at 23 and 27 for pre-existing diabetes and GDM respectively, leaving it underpowered. Its glycosuria spectrum was larger and better powered, but glycosuria is an indirect marker of maternal hyperglycemia and less specific to the exposures of interest.

Aside from metabolic profiles and blood pressure, prenatal glycemic status appears to influence cardiovascular stress responses and arterial stiffness. Offspring of mothers with gestational diabetes were found to demonstrate greater cardiosympathetic responses, stroke volume, and stress-induced SBP during psychological challenges compared to offspring of non-diabetic women (88). Cord C-peptide and insulin levels at birth, downstream biomarkers of intrauterine glycemic exposure, were both positively correlated with increased arterial stiffness (96), though the strength of these associations may be age-dependent, as data suggest the relationship between cord blood insulin and blood pressure is prominent in childhood but becomes non-significant by late adolescence (51).

The mechanisms linking maternal hyperglycemia to offspring cardiovascular dysfunction center largely on the fetal programming of metabolic and endocrine systems. In utero exposure to high glucose levels triggers fetal hyperinsulinemia, which may alter the development of the hypothalamus. This disruption can lead to a reprogramming of the HPA axis and the autonomic nervous system, and has been proposed as an explanation for the heightened cardiosympathetic reactivity observed in adolescence (88). Additionally, the anabolic properties of fetal hyperinsulinemia and potential leptin resistance may alter hypothalamic appetite regulation, driving the increased food intake and obesity that may mediate the relationship between GDM and elevated SBP (49, 88). The adverse lipid profile observed may similarly reflect metabolic reprogramming. Altered maternal lipid metabolism may then alter offspring lipid metabolism; animal models associate maternal hyperglycemia and altered offspring hepatic lipid content through oxidative stress and inflammation (49).

Gestational hyperglycemia may also impair the function of specific organ systems. Gestational hyperglycemia is thought to affect nephron development and potentially lead to reduced nephron mass. This reduction can trigger glomerular hyperfiltration in the remaining units, as evidenced by the higher eGFRs seen in offspring of T1DM mothers, and lead toward hypertension and progressive renal dysfunction later in life (74). Nonetheless, the extent to which these direct programming pathways independently contribute to adolescent cardiovascular risk beyond their influence on offspring adiposity remains to be clarified.

### 3.7 Maternal obesity

Maternal pre-pregnancy obesity, one of the most prevalent modifiable risk factors in pregnancy, is consistently associated with adverse cardiovascular outcomes in adolescent offspring, including significantly higher SBP and impaired glucose metabolism (39, 52).

Several intrauterine signaling pathways are proposed to drive this association. Increased fetal exposure to maternal lipids and inflammatory cytokines may dysregulate the HPA axis and induce epigenetic changes (39). Additionally, maternal fat deposition may enhance the placental transfer of nutrients, potentially programming the fetus for metabolic dysfunction (52). The significance of the intrauterine environment is supported by sibling studies; children born after their mother underwent weight-reducing gastric bypass surgery show marked improvements in insulin sensitivity and adiposity compared to their siblings born before the surgery, which were sustained into adolescence (53). Beyond endocrine and metabolic signaling, structural developmental changes in the renal system may occur and predispose the offspring to cardiovascular risk. Maternal obesity is associated with a reduction in late-gestational fetal kidney volume in proportion to total fetal body weight (53). Given that kidney volume is considered a proxy for nephron number, these structural deficits may constitute a risk factor for chronic kidney disease and systemic hypertension later in life.

However, epidemiological studies indicate that the relationship between maternal obesity and offspring SBP is heavily dependent on childhood outcomes. The association with increased adolescent blood pressure and adverse cardiometabolic profiles is almost entirely attenuated when adjusting for the adolescent's current weight status, and notably, adjusting for birth weight does not explain this relationship (29, 39, 52). Longitudinal data reveal that concurrent BMI at age 17, rather than the overall BMI trajectory throughout adolescence, serves as the superior predictor of blood pressure (39). Ultimately, this suggests that while maternal obesity can predispose offspring to adverse cardiovascular outcomes, this risk appears to be primarily mediated by the offspring's weight status during adolescence rather than direct fetal programming.

### 3.8 Nutritional factors

Nutritional supplementation during pregnancy is generally not associated with significant changes in adolescent blood pressure, although it may exert a subtle influence on select cardiovascular and metabolic markers (75). Human trials suggest that the cardiovascular system is relatively resilient to specific isolated nutritional interventions (76).

The primary motivation for macronutrient supplementation is to prevent the development of a thrifty phenotype, where poor fetal nutrition is hypothesized to program offspring for insulin resistance and fat storage (75). Because insulin is a recognized risk factor for arterial stiffness, adequate protein and calorie intake during development is thought to protect vascular elasticity. Human trials partially support this notion. In a trial in the Gambia, where undernutrition is common, protein and calorie supplementation during pregnancy was associated with a 3.3% lower augmentation index and 20% lower homeostatic model assessment of insulin resistance in adolescent offspring. However, despite these favorable vascular findings, studies found little effect of nutritional supplementation on blood pressure (75, 77). Interestingly, nutritional supplementation was associated with increased blood pressure in adolescents in the lowest quartile for body fat (77). One proposed explanation for this discrepancy is a prenatal-postnatal mismatch. If supplementation programmed the fetus to expect a more nutrient-rich environment than the child ultimately experienced after birth, the resulting mismatch could itself promote cardiovascular risk, suggesting that postnatal nutrition may have a more significant impact on blood pressure than prenatal supplementation alone.

Vitamin D has been implicated in the regulation of cardiovascular physiology influencing myocyte proliferation, hypertrophy, and the renin-angiotensin system. It also contributes to healthy placentation and angiogenesis, which are the foundations of fetal vascular development (54). However, studies show that maternal vitamin D levels have no significant association with offspring blood pressure or PWV in the general adolescent population (78, 79). A potential exception occurs in high-risk pregnancies, where it may exert a protective effect. In children born to mothers with PE, higher cord blood vitamin D levels are associated with lower SBP (54). Similarly, long-chain n-3 polyunsaturated fatty acids contribute to vascular development in animal models, where deficiency leads to increased blood pressure. In human trials however, maternal fish oil supplementation during the third trimester did not result in significantly lower blood pressure in offspring evaluated at 19 years of age (76).

Calcium is essential during pregnancy for the mineralization of the fetal skeleton and the foundational development of the fetal vascular system, and improved maternal calcium levels may improve fetal vascular smooth muscle function and autonomic regulation. Research has shown that in areas with low customary calcium intake (300–400 mg/d), female offspring born to mothers receiving calcium supplementation exhibited significantly lower SBP at all ages compared to controls, with a mean reduction of 2.1 mmHg (55). However, this cardiovascular benefit may not reflect a direct vascular effect alone. Girls in the calcium group also demonstrated a 3% lower peak height velocity than the placebo group, and because pubertal growth velocity is positively associated with blood pressure, the observed SBP reduction may be partly mediated through altered growth tempo rather than vascular programming. While the exact mechanism remains unknown, it is likely mediated by sex-specific changes in the growth hormone to IGF1 axis, with calcium exposure leading to lower plasma IGF1 concentrations in girls, a pattern that was not found in boys.

As a whole, the limited and inconsistent effects of individual nutritional interventions suggest that the prenatal programming of adolescent blood pressure is unlikely to be driven by any single dietary factor in isolation.

### 3.9 Environmental exposures

Prenatal environmental exposures show varied associations with adolescent cardiovascular outcomes. Maternal smoking and certain chemical pollutants, including persistent organic pollutants (POPs) and per- and polyfluoroalkyl substances (PFAS), show consistent associations with elevated blood pressure and metabolic risk, while other exposures such as mercury, noise, and phthalates show little evidence of long-term effects on cardiovascular outcomes.

The primary hypothesized mechanism for the cardiovascular impact of maternal smoking is fetal growth restriction, as low birth weight has consistently been associated with hypertension and cardiovascular disease later in life. Other proposed pathways include increased arterial resistance, endothelial dysfunction, and structural alterations in both the kidneys and perivascular adipose tissue (56). Clinical data indicate that maternal smoking is significantly associated with small but consistent increases in offspring blood pressure, often showing a more pronounced effect on diastolic than systolic values (30, 56). Notably, smoking cessation at any point during pregnancy does not appear to mitigate these adolescent cardiovascular risks compared to those who never smoked, suggesting a critical early intrauterine effect (30).

POPs are hypothesized to act through endocrine disruption, specifically androgen and estrogen receptor antagonism, which may interfere with energy homeostasis and adipocyte differentiation (57, 58). Exposure to POP mixtures, particularly hexachlorobenzene and dichlorodiphenyldichloroethylene, is linked to higher BMI and increased SBP in girls. PFAS have also been implicated in cardiovascular programming through proposed mechanisms including oxidative stress, mitochondrial dysfunction, and decreased renal nephron endowment (31). Human observational data show that higher maternal concentrations of certain PFAS are associated with higher SBP percentiles, with these associations appearing stronger in male and Black children.

In preclinical models, heavy metals, particularly lead, induce oxidative stress, decrease nitric oxide availability, and disrupt calcium transport and distribution (32). In a cohort study in Greece, lead and molybdenum were the components most consistently associated with blood pressure elevations in late childhood and adolescence (32), whereas, a UK study found little evidence suggesting that prenatal mercury exposure has significant adverse long term effects on blood pressure (59).

Similarly, prenatal exposure to noise, phthalates, and phenols has shown little association with adolescent blood pressure or heart rate (33, 80). Overall, while maternal smoking and certain chemical pollutants appear to program lasting cardiovascular changes, the inconsistency across exposure types underscores that the nature, timing, and dose of the environmental insult likely determine whether a durable effect on adolescent cardiovascular outcomes is established.

### 3.10 Other factors

Beyond the major exposure categories discussed above, a smaller body of literature has examined how less commonly studied prenatal exposures, including substance use and severe pregnancy-related nausea, influence the adolescent stress response and blood pressure.

Prenatal drug exposure is increasingly linked to alterations of the HPA axis, which governs the body's response to stress. Specifically, adolescents with prenatal cocaine exposure exhibit significantly higher salivary cortisol levels during stress tests compared to their unexposed counterparts (84). While heart rate differences may not be significant, these elevated cortisol levels suggest that early alterations in HPA axis functioning persist into adolescence. This shift in developmental trajectory may result from direct drug exposure, the chronic stress often experienced in these environments, or a genetic predisposition to HPA axis disruption (84).

The impact of prenatal substance exposure can also manifest in unexpected ways within specific demographic groups. Research involving Hispanic adolescents found that those exposed to a singular substance prenatally had an SBP that was 13 mmHg higher at age 16 than unexposed peers (60). Interestingly, those exposed to polysubstance use (two or more substances) exhibited a paradoxical trend where SBP continuously decreased as the number of substances increased, remaining within a healthy range (60). This phenomenon is hypothesized to be linked to the “Hispanic paradox,” where cultural and community factors, such as dietary habits, foreign-born status, or the “Barrio advantage,” may serve as protective mediators against adverse cardiovascular outcomes despite adverse early exposures (60).

In contrast to the significant impacts of substance exposure and hypertensive disorders, other severe pregnancy-related conditions show little evidence of long term cardiovascular programming. Hyperemesis gravidarum, despite its physiological severity for the mother during pregnancy, has no significant association with adolescent SBP (61).

### 3.11 Strengths and limitations of evidence

The consistency of associations between prenatal exposures and adolescent cardiovascular outcomes varies substantially across exposure-outcome pairs (Table 3). Blood pressure is the most frequently studied outcome and shows the highest proportion of positive findings for HDP (15 of 16 studies), birth weight (11 of 14), and preterm birth (10 of 12). Cardiac function outcomes, though less frequently assessed, are consistently associated with birth weight and preterm birth (6 of 6 and 6 of 9 studies, respectively). In contrast, cardiometabolic risk factors show a more mixed pattern across all exposure categories, with near-equal proportions of positive and null findings for birth weight (4 of 8) and maternal diabetes (7 of 12). Arterial stiffness is the least studied outcome overall, with no exposure category supported by more than four studies.

**Table 3:** Principal cardiovascular outcomes of each prenatal exposure.

Exposure category	Outcome category	Studies (*n*)	Positive findings	Null findings
Preterm birth	Cardiometabolic risk factors	5	2	3
	Cardiac function	6	6	0
	Blood pressure	12	10	2
	Arterial stiffness	4	2	2
Birth weight	Blood pressure	14	11	3
	Cardiac function	9	6	3
	Cardiometabolic risk factors	8	4	4
Environmental exposure	Arterial stiffness	3	1	2
	Blood pressure	12	8	4
	Cardiac function	1	0	1
	Cardiometabolic risk factors	8	7	1
Hypertensive disorders of pregnancy	Blood pressure	16	15	1
	Cardiac function	3	2	1
	Cardiometabolic risk factors	12	8	4
Maternal diabetes	Arterial stiffness	1	1	0
	Blood pressure	8	5	3
	Cardiac function	1	1	0
	Cardiometabolic risk factors	12	7	5
Maternal obesity	Blood pressure	5	4	1
	Cardiometabolic risk factors	7	6	1
Nutrition	Arterial stiffness	2	1	1
	Blood pressure	7	2	5
	Cardiac function	2	0	2
	Cardiometabolic risk factors	2	1	1
Other	Blood pressure	4	3	1
	Cardiac function	1	0	1
	Cardiometabolic risk factors	1	1	0

Positive and null findings were determined during full-text review of each study. A finding was classified as positive if the study reported a statistically significant difference in the outcome of interest between the exposed and the comparison group; otherwise, it was classified as null. Studies frequently reported multiple outcomes of interest, and each outcome was classified independently. Counts therefore represent the number of exposure-outcome associations and not the number of studies. These totals reflect the number and direction of reported associations only and do not represent a formal grading of evidence quality, effect magnitude, or precision.

Several methodological limitations limit the strength of evidence. Outcome measurement varies widely across studies; blood pressure is assessed by office readings, ambulatory monitoring, or exercise response, and arterial stiffness by carotid-femoral, carotid-radial, or MRI-derived indices, each capturing different vascular segments and properties. These differences complicate direct comparison across cohorts, as discussed in the preterm birth literature where four studies using four different stiffness methods produced conflicting results (42, 45, 72, 73). Furthermore, sex stratification was inconsistently applied across studies, limiting the conclusions that can be drawn about sex differences.

Confounding factors also remain incompletely addressed across the literature. Genetic predisposition is rarely disentangled from intrauterine programming, and several authors have noted that shared maternal-child genetic variants could independently predispose both to the prenatal exposure and to the offspring cardiovascular outcome, as proposed for HDP-associated cardiac remodeling and maternal diabetes-associated cardiometabolic risk (27, 50, 89). Socioeconomic position is associated with both prenatal exposures and offspring cardiovascular risk, yet adjustment for socioeconomic factors is inconsistent across studies. Lifestyle factors, particularly diet and physical activity, are sometimes not adjusted for and typically self-reported when available, as seen in the maternal diabetes literature (49, 50, 74). Given these limitations, the independent contribution of intrauterine programming remains uncertain, particularly for exposures where these confounders are most plausible as alternative explanations.

Finally, this narrative review has limitations related to its search and review methodology. The search was limited to one database, PubMed, and to English literature only, and the literature search and data extraction were conducted by a single reviewer. In addition, a formal risk-of-bias assessment was not undertaken, and the strength of studies was not systematically graded. These limitations should be considered while interpreting the conclusions of this review.

## 4 Conclusion

Current literature suggests that adolescent cardiovascular outcomes may be explained partially by a complex clustering of prenatal exposures rather than any single isolated factor. Growth-related metrics, specifically preterm birth and low birth weight, emerge as the most consistent correlates of elevated SBP and distinct structural phenotypes, such as aortic narrowing and reduced ventricular chamber size. These findings highlight a critical developmental window where disrupted gestation leads to cardiovascular alterations that may persist into adulthood.

Beyond growth metrics, maternal health conditions like PE and GH demonstrate strong associations with adverse cardiac remodeling and altered endocrine regulation. Similarly, maternal obesity and diabetes correlate with adverse lipid profiles and increased adiposity in adolescence. However, in contrast to the structural differences associated with growth restriction, the cardiovascular risks associated with maternal obesity and hyperglycemia appear to be largely mediated by current offspring BMI. This raises the possibility that shared genetics, lifestyle, and postnatal environmental factors may have a larger impact than previously appreciated on these specific outcomes.

In contrast, prenatal nutritional interventions generally show limited associations with adolescent blood pressure, suggesting that postnatal nutrition and catch-up growth trajectories may exert a more significant influence than prenatal nutrition. An exception is vitamin D, which may serve a protective role against the hypertensive effects of PE. Beyond nutrition, environmental stressors, particularly maternal smoking, persistent organic pollutants, and heavy metals, are associated with elevated long-term risk, potentially through pathways such as oxidative stress and impaired organogenesis.

Several mechanistic themes are common among prenatal exposures (Figure 1). The proposed mechanisms of the programming of cardiovascular vulnerability often converge on impaired renal development, disrupted autonomic regulation, and HPA axis dysregulation. These early intrauterine shifts may create a physiological baseline that predisposes individuals to cardiovascular and metabolic disease. Ultimately, the intrauterine environment serves as a foundational period for heart and vascular development during which prenatal stressors interact with postnatal growth patterns in shaping an individual's long-term cardiovascular health.

**Figure 1:**
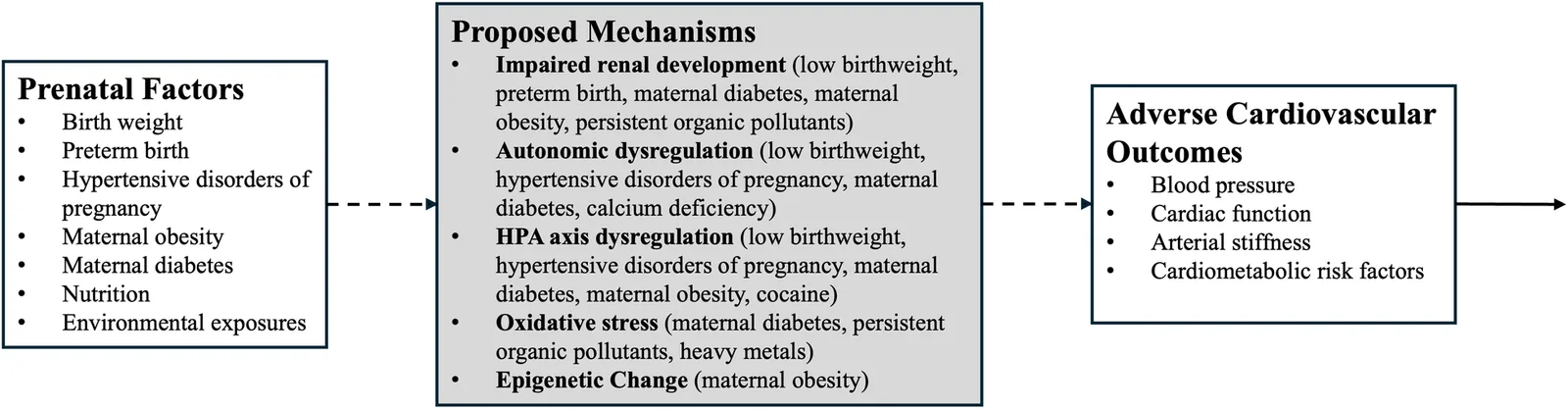
Proposed mechanisms that may contribute to prenatal programming of adolescent cardiovascular risk. Dashed arrows indicate hypothesized associations and candidate mechanistic pathways derived largely from observational studies; they do not denote established causal relationships. The relative contribution of each pathway remains uncertain.

### 4.1 Clinical implications

The findings of this review suggest prenatal history may be a useful tool for identifying adolescents who warrant closer monitoring for cardiovascular risk. The AAP Clinical Practice Guideline for high blood pressure in children already recommends obtaining perinatal history, including maternal pregnancy complications, gestational age, and birth weight, as part of the diagnostic evaluation for hypertension (107). The evidence synthesized here reinforces that recommendation and suggests specific prenatal exposures that may inform risk detection.

Low birth weight and preterm birth carry the most consistent evidence linking prenatal adversity to elevated adolescent blood pressure and adverse cardiac structure. Former preterm infants have been shown to display unfavorable cardiovascular risk profiles as early as preschool age, and the AHA has identified premature birth among the growing list of pediatric conditions associated with accelerated atherosclerosis (108). Adolescents with these histories may benefit from more vigilant blood pressure surveillance and earlier lifestyle counseling, though formal screening protocols have not been established. Maternal HDP represent another exposure with strong and consistent associations with offspring blood pressure. Notably, current postpartum guidelines focus on the mother's long-term cardiovascular risk after HDP, but less attention is given to the offspring (109). Integrating maternal HDP history into the adolescent's medical record could facilitate targeted blood pressure monitoring, but this suggestion would require additional evaluation before adoption. The clinical picture for maternal diabetes and obesity is more nuanced. Because the associated cardiovascular risks are largely mediated by offspring adiposity, weight management during childhood and adolescence may be the most effective intervention. Overall, a prenatal history-informed approach to adolescent cardiovascular risk does not require new screening methods but rather the integration of perinatal data, already collected at birth, into pediatric care.

### 4.2 Future directions

Despite the breadth of studies investigating prenatal factors and adolescent cardiovascular outcomes, the links between many exposures and long-term outcomes remain incompletely understood. The most robust evidence centers on growth related metrics (birth weight, preterm birth) and maternal cardiometabolic conditions (PE, diabetes), whereas environmental exposures, including nutritional factors, persistent organic pollutants, PFAS, and heavy metals, are comparatively understudied (110). The American Heart Association has highlighted that the vast majority of the estimated 300,000 synthetic chemicals registered for international use have not been evaluated for pediatric cardiovascular effects, and most existing studies assess single exposures rather than the complex mixtures encountered in real life (111). Similarly, prenatal nutritional interventions have shown limited cardiovascular effects in human trials despite strong signals in animal models (112–114), suggesting that the relevant exposure windows, nutrient interactions, and dose-response relationships require further study (110).

Multi-omics offers a promising approach to bridge these gaps. Metabolomics, an integrated readout of genetic, epigenetic, and environmental variation, can capture the biological imprint of diverse prenatal exposures in a single analytical platform (115, 116). Early cord blood metabolomics studies have already identified acylcarnitines, purine metabolites, and branched-chain amino acids as prospective predictors of childhood and adolescent blood pressure and insulin resistance (117). More recent work has linked cord blood metabolomic signatures to specific prenatal exposures, including metals and PFAS, identifying disrupted pathways in fatty acid oxidation, amino acid metabolism, and oxidative stress defense that may mediate downstream cardiometabolic risk (118, 119). The HELIX (Human Early Life Exposome) project has demonstrated the feasibility of associating over 100 prenatal and childhood exposures with multi-omics profiles, finding that pregnancy exposures are predominantly associated with offspring DNA methylation changes while childhood exposures more frequently alter the serum metabolome.

Beyond metabolomics, epigenomic profiling may help clarify how prenatal exposures become biologically embedded. DNA methylation changes at birth have been associated with several of the exposures reviewed here, including maternal smoking, HDP, and fetal growth restriction (120, 121), and may serve as molecular mediators linking the intrauterine environment to downstream cardiovascular outcomes. Transcriptomic and proteomic analyses of cord blood or placental tissue could further map the gene expression programs altered by specific prenatal stressors, though these approaches remain largely unexplored in adolescent cardiovascular cohorts. Integrating multi-omics within existing birth cohorts would better capture the complexity of prenatal programming and help clarify the specific biological pathways through which understudied prenatal exposures, particularly environmental chemicals and nutritional factors, program the adolescent cardiovascular outcomes described in this review.

If these multi-omics approaches can identify biosignatures of prenatal cardiovascular programming, they may ultimately support more precise early risk prediction. Rather than relying solely on conventional risk factors ascertained in adolescence, composite profiles incorporating prenatal exposures, perinatal omics data, and postnatal growth trajectories could enable more individualized identification of at-risk youth.
