# Supplementary material for: Prenatal factors shaping adolescent cardiovascular function and cardiometabolic risk trajectories
Source: Front Pediatr. 2026 Sep 14;14:1933223. doi: 10.3389/fped.2026.1933223 (PMC13617285; doi:10.3389/fped.2026.1933223)
Supplement: Supplementary file 1 [file Table1.docx]

| **Table S1. PubMed Search String** | | |
| --- | --- | --- |
| **Set** | **Concept** | **Keywords** |
| 1 | Prenatal exposures | "Prenatal Exposure Delayed Effects"[MeSH Terms] OR "Fetal Development"[MeSH Terms] OR "Maternal Exposure"[MeSH Terms] OR "Pregnancy Complications"[MeSH Terms] OR "fetal programming"[Title/Abstract] OR "developmental origins"[Title/Abstract] OR "in utero exposure*"[Title/Abstract] OR "prenatal factor*"[Title/Abstract] OR "prenatal exposure*"[Title/Abstract] OR "DOHaD"[Title/Abstract] |
| 2 | Cardiovascular outcomes | "Cardiovascular Physiological Phenomena"[MeSH Terms] OR "Cardiovascular Diseases"[MeSH Terms] OR "Vascular Stiffness"[MeSH Terms] OR "Blood Pressure"[MeSH Terms] OR "Heart Rate"[MeSH Terms] OR "Cardiac Output"[MeSH Terms] OR "Echocardiography"[MeSH Terms] OR "vascular function"[Title/Abstract] OR "cardiovascular function"[Title/Abstract] OR "arterial stiffness"[Title/Abstract] OR "flow mediated dilation"[Title/Abstract] OR "pulse wave velocity"[Title/Abstract] OR "endothelial function"[Title/Abstract] OR "cardiac remodeling"[Title/Abstract] |
| 3 | Adolescents | "adolescent"[Title/Abstract] OR "adolescence"[Title/Abstract] OR "teen*"[Title/Abstract] |
| 4 | Humans | humans [MeSH Terms] |
| 5 | Combined | #1 AND #2 AND #3 AND #4 |
